# Discursive Analysis of Pediatrician’s Therapeutic Approach towards Childhood Fever and Its Contextual Differences: An Ethnomethodological Study

**DOI:** 10.3390/children11030316

**Published:** 2024-03-07

**Authors:** Francisco Vicens-Blanes, Rosa Miró-Bonet, Jesús Molina-Mula

**Affiliations:** Departamento de Enfermería y Fisioterapia, University of Balearic Islands, 07122 Palma, Spain; rosa.miro@uib.es (R.M.-B.); jesus.molina@uib.es (J.M.-M.)

**Keywords:** pediatrician, children, fever

## Abstract

**Introduction:** Fever stands out as the predominant clinical indicator in infancy. Pediatricians encounter fever routinely in their daily practice, playing a crucial role in their interactions with children and families. **Objective:** The aim is to examine pediatricians’ viewpoints, understanding, and approaches regarding childhood fever in two healthcare settings: pediatric hospitalization (emergency and inpatient ward) and primary care. **Methods:** A qualitative study was conducted using an ethnomethodological approach. Pediatricians working in the specified pediatric settings participated in in-depth interviews where theoretical clinical cases were presented for analysis. **Results**: Following the examination of the discourses, the codes were organized into eight categories: Understanding of fever, Significance ascribed to fever, Therapeutic strategies, Engagement with the evidence, Family apprehensions regarding fever, Influence of the COVID-19 Pandemic, Inter- and intra-professional relationships, and Suggestions for change: **Conclusions:** Pediatricians acknowledge the importance of addressing discomfort in the treatment of fever, but express challenges in implementing these recommendations. Pediatricians in inpatient settings emphasize the need for enhanced parental education from primary care, while those in primary care recognize the potential for improvement. Inpatient pediatricians are open to implementing changes in their daily practices, particularly concerning the administration of antipyretics.

## 1. Introduction

Episodes of fever in children create undue distress and place a significant emotional strain on families. They are the primary reason for seeking healthcare through primary care or emergency hospital services [1]. The period between 3 and 36 months of age marks the peak incidence phase for febrile episodes in the pediatric stage [1,2].

A child’s body temperature increase typically arises from an underlying infection. Fever, the body’s immunological and physiological response to the infection, is commonly viewed as potentially harmful and perilous for children [3,4,5].

The apprehensions and fears regarding fever are not limited to parents; as the published literature indicates, healthcare professionals frequently express their concerns through their attitudes toward children with fever [6,7].

Following the National Institute for Health Care Excellence (NICE) guidelines, fever management should not solely concentrate on reducing the temperature. Rather, it should also strive to alleviate discomfort [8].

Pediatricians and pediatric nurses serve as the primary sources of information for parents regarding concepts related to fever and its management. The messages they communicate, along with the observations parents make of their actions, play a crucial role in influencing how families handle their child’s febrile episodes [3,7,9].

Healthcare professionals frequently take action to reduce a child’s temperature due to concerns about potential complications, lack of awareness of recommendations, or adherence to professional requirements. In pursuing this objective, they may explicitly or implicitly communicate to parents that fever is harmful and emphasize the urgency of achieving normal body temperature [10,11].

Certain authors emphasize the importance of professional training and how a lack of up-to-date knowledge can lead healthcare professionals to approach a febrile child with anxiety with a primary focus on reducing temperature [3,4,12]. Furthermore, some authors propose that cultural factors heavily influence the actions taken during the care or treatment of a febrile child. They suggest that certain professional attitudes may align more with traditional beliefs than practical knowledge [13,14].

Taking these considerations into account, pediatricians must provide parents with the necessary information to assess signs and symptoms of potentially serious underlying illnesses. The emphasis should be on addressing the child’s discomfort rather than solely concentrating on reducing temperature [15].

Research investigating pediatricians’ perceptions, knowledge, and attitudes toward childhood fever is predominantly descriptive. As an example, the results of a questionnaire distributed to Italian pediatricians reveal that more than one-third of them (38.2%) would base their approach to temperature treatment on addressing the child’s discomfort [15].

Given the considerations described earlier, this study aims to assess pediatricians’ perceptions, knowledge, and attitudes concerning childhood fever in both hospital and primary care settings.

## 2. Methodology

### 2.1. Design

This study adopts a qualitative approach within the constructivist paradigm, employing an ethnomethodological framework. The primary goal is to analyze pediatricians’ perceptions, knowledge, and attitudes regarding childhood fever.

An ethnomethodological approach is deemed an optimal qualitative research method as it investigates how social group members perceive, define, and categorize the activities conducted in their daily lives, along with the meanings they attribute to these activities. This approach concentrates on aspects of social life that are so commonplace and deeply embedded that deliberate reflection is required to comprehend their significance and appreciate their importance [16,17].

For pediatricians, managing fever is an integral part of their daily routine, and its frequent occurrence may result in it being overlooked due to the high degree of repetition. This can diminish the emphasis on its significance, and certain activities may become automated without thoughtful reflection. Hence, ethnomethodology enables a concentrated examination of this routine activity, facilitating reflection, assessment of actions within the daily practice of these professionals, and a deeper exploration of the factors influencing their actions.

### 2.2. Theoretical Perspective

To articulate the findings of this study, we suggest adopting a theoretical pluralism that aligns with the analysis. Harper’s theoretical perspective is employed in this context to elucidate the fundamental concepts of ethnomethodology and tailor them to the current phenomenon [17]. Additionally, to attain the interpretative depth necessary for a study of this nature, elements from the works of Freire, Bandura, and Bolívar were incorporated to rigorously frame the discussion of the results [18,19,20].

### 2.3. Contextualization

The Balearic Islands community in Spain comprises three health areas: Mallorca, Menorca, and Ibiza and Formentera. The Mallorca area is further divided into four sectors.

The fieldwork for this study is centered around the Migjorn sector of the Mallorca area. This sector has a population of approximately 270,276 people, with a pediatric population constituting 15.61%, equivalent to around 42,190 inhabitants aged between 0 and 14 years old.

This health sector comprises basic health zones, with 14 health centers forming these zones.

Within this health center are around 2 doctors in the pediatrics offices. These doctors may specialize in pediatrics, or they could be family doctors providing care for children.

The Migjorn health sector designates the Son Llàtzer Hospital in Palma de Mallorca as its reference hospital.

The hospital has a total of 27 attending physicians and 14 residents who specialize in pediatrics. It is noteworthy that a considerable number of the affiliated pediatricians work reduced hours.

### 2.4. Participants

The research was conducted at a tertiary-level hospital, specifically, the Hospital Universitario Son Llàtzer, situated in Palma de Mallorca, Spain, along with the 14 health centers within its reference area. The contexts of pediatric hospitalization, pediatric hospital emergency, and primary care were explored by the authors as part of their study.

Eleven pediatricians whose professional responsibilities were relevant to the contexts under examination and who had expressed interest in participating were recruited for this study. These physicians were approached through a key informant. Subsequently, the principal investigator contacted the interested physicians via e-mail or through a messaging application.

The principal investigator is a specialized pediatric nurse committed to education and research in pediatrics.

The snowball strategy was implemented when an interviewed participant informed us that another service professional was interested in participating.

The interview location varied based on the context being studied. For hospital pediatricians, the interviews took place in a room used for clinical sessions and an unoccupied observation room in the same unit. In the case of primary care pediatricians, the interviews were conducted in their respective consultation rooms.

The initial interview occurred on 30 May 2022, and the final on 15 July 2022. Among the participating physicians, six were from hospital settings and five were from primary care.

The inclusion criteria for participants were as follows:

- Pediatricians eligible for participation included those actively working in the pediatric inpatient ward, pediatric emergency unit, and health centers affiliated with the area of the hospital indicated above.
- In addition, the general physician provides care to children in health centers affiliated with the area of the hospital indicated above.

The exclusion criteria for participants were as follows:

- Exclusion criteria involved individuals not actively engaged in professional activities within one of the studied contexts.

Pediatricians employed in the hospital typically have a primary area of focus, such as the emergency department or the hospital ward. Additionally, they are often assigned 24 h on-call duties to attend to children from both departments.

### 2.5. Data Collection and Analysis

This study employed two data collection techniques: interviews and the field diary maintained by the principal investigator.

Initially, data collection involved a conversational technique utilizing semi-structured in-depth interviews. These interviews employed a guide consisting of open-ended questions and lasted between 30 min and 1 h. The interview guide can be found in the Supplementary Material of this article.

Discourse refers to the use of language as a form of social practice. Analysis discourse enables the researcher to concentrate on the meanings conveyed by participants’ communication and interpret these meanings in the context of social and cultural influences and customs [21].

At the outset of the interviews, participants were queried for the following socio-demographic information: their department of employment, age, gender, specialty, tenure in pediatric services, parental status, membership in any pediatric-related scientific association, and whether they held any managerial positions (Table 1 and Table 2).

The age of hospital pediatricians varies between 32 and 56 years, with their work experience spanning from 4 to 30 years. These pediatricians are members of a scientific organization, although none holds a management position. Only one participant among the hospitalization staff has children.

Primary care pediatricians are aged 33 to 64 years, with work experience ranging from 13 to 34 years. Like hospital pediatricians, all primary care pediatricians are affiliated with a scientific organization, and one holds a management position. Notably, four of the primary care participants have children.

During the interview process, a set of infographics was presented, incorporating the following recommendations:

- The “Decalogue of Fever” from the Spanish Association of Primary Care Pediatrics (2011), designed for family members, was included among the infographics during the interview.
- The infographics also featured “Fever in under 5s: Assessment and initial management” from the NICE Guidelines (2019), intended for healthcare professionals.

Concluding the interview, two clinical cases were presented, illustrating common situations encountered in clinical practice involving a febrile child. The content of these cases was tailored to align with the pediatric context of each interviewed pediatrician.

Secondly, the principal investigator kept a field diary throughout the research. This diary comprises written or audio-recorded notes taken before or after the interviews. It includes observations and self-observations that have prompted thoughts, emotions, or ideas contributing to the external and internal validity of the study [22].

Building upon the entries in the field diary, adjustments were made to the clinical cases presented in the interviews to garner more insights from the daily practices of pediatricians. Changes were also implemented in the form and timing of presenting evidence recommendations to alleviate tensions observed in initial interviews. Certain questions were modified, incorporating new ones that emerged as intriguing topics during the preliminary analysis, contributing to a more comprehensive exploration of the study phenomenon.

The data followed a cyclical process involving inductive and deductive analyses, following several phases [21].

Initially, the information for analysis was gathered through in-depth interviews and notes from the principal investigator’s field diary. The interviews were recorded using an audio recorder and later transcribed using the online tool “Sonix”. Each transcript underwent a thorough review by the principal investigator.

In the second phase, the obtained texts were anonymized, and acronyms were assigned for each context, along with a number for each interview conducted. The texts were read, and initial annotations were made, including underlining and comments on the most significant sections. Following multiple readings, the gathered information was systematically coded.

An inductive analysis was conducted from the transcribed texts, encoding the information from interviews or observations by highlighting words, phrases, or entire paragraphs to identify specific topics and ascertain their significance. Following this, a deductive analysis was undertaken to understand the patterns in the data and attribute meaning to the identified categories [21].

In the cyclical analysis process, the initial phase involved a descriptive examination of the data and codes to establish the groundwork for the subsequent interpretative phase. Ultimately, the data were organized into categories and codes, synthesizing the information and extracting meanings, comparisons, and conclusions within an ethnomethodological framework.

The coding trees in Figure 1, Figure 2, Figure 3, Figure 4, Figure 5, Figure 6, Figure 7 and Figure 8 visually represent the analyzed categories and codes.

Each context interview was shared with various research team members, including professionals from the studied environment.

### 2.6. Ethical Considerations

Participating pediatricians were provided with comprehensive information both verbally and in writing through an information sheet. Before the interviews, each participant read and signed the informed consent form. The information and data collected from the participants were anonymized and securely stored by the study’s principal investigator.

This study received approval from the Research Ethics Committee of the Balearic Islands (CEI-IB) on 5 November 2021. Additionally, it was sanctioned by the ethics committees of both Son Llàtzer University Hospital and the Primary Care Management of Mallorca.

### 2.7. Results

This section presents the study’s results organized into different categories, accompanied by each case’s most significant textual discourses. These are coded to ensure participant anonymity. However, the coding enables the identification of the participant’s context, aiding the comprehension of the results (P represents primary care and H represents hospital care; when a “C” is added, it refers to the response given to a case).

### 2.8. Concept of Fever

This category encompasses discourses arising from pediatricians’ responses regarding fever in children. The definition of fever, as articulated by pediatricians, is often associated with a specific temperature threshold commonly utilized to determine the presence or absence of fever. The statements reveal that some participants prefer employing biochemical and physiological terms to describe the processes that lead to an increase in a child’s temperature.

Participants generally perceive fever as a defense or warning mechanism of the body, primarily against infections. Their statements also highlight a conflict between traditional perspectives on fever and more contemporary information, particularly regarding whether to address the temperature or the discomfort it causes.

*Fever is an increase in body temperature in relation to the body’s struggle to defend itself against some process of aggression, whether physical, infectious, autoimmune, inflammatory, etc.*

P4

*I understand a fever to be a rectal or axillary temperature above 38 °C, and it is recorded by a validated thermometer.*

H1

*The objective (of antipyretics) is to remove the discomfort it (the fever) generates. Although I think children tolerate it (fever) much better.*

P2

*Regarding the time of the visit 37.6 (°C), there’s one thing that I think is right the nurse says, that you won’t be administered until you’re 38 (°C). That makes sense in terms of infection, doesn’t it? We consider a fever to be 38 (°C) or higher, so if the fever itself does not reach 38 (°C), we do not consider it a fever. I’m fine with waiting for 38 (°C) because that does change my therapeutic attitude.*

H2C2

### 2.9. Importance Attributed to Fever

The category of importance attributed to the phenomenon of fever in children encompasses the discourses of participating physicians that explicitly or implicitly convey the impact of caring for febrile children on their professional activity.

Certain pediatricians emphasize the significance of the phenomenon of fever due to factors such as the number of emergency room visits it prompts, the anxiety it induces in parents, and the perceived necessity for professionals to reduce the child’s temperature.

On the other hand, some discourses assert that professionals generally face no difficulties handling fever in children, and parents swiftly adapt to its management. Concerning the elevated number of visits stemming from fever and the perceived lack of internalization of information by families, certain pediatricians express frustration with the constant demand for attention and management of these cases, both by themselves and their colleagues.

*I try to convey to the parents that “Fever is important, it’s good that the child has a fever now.” I try to get them to understand that the fever has a function for a parent. It is fundamental because the anxiety that the fever causes is going to change.*

H1

*I think so (fever is a problem) because it is the main reason for consultation in the emergency department. So it is always a cause of stress (…) Fever causes intense stress to. It is the main reason for consultation in the emergency department, to the pediatrician, to 061, (Ambulance service) to everybody. It is very stressful.*

H4

*I think they receive little information about fever because of the care load and because it is such a frequent reason for consultation that pediatricians are tired of repeating it. Sometimes there are mornings, afternoons, or evenings on call when you see patients who have the same symptoms, and you are very tired and it becomes routine. It becomes very routine and stops arousing your interest.*

H1

### 2.10. Therapeutic Approach

This category encompasses discourses on the routine therapeutic approach adopted for a febrile child, with pediatricians prioritizing conveying appropriate messages. Interestingly, participants acknowledge that families may perceive certain inconsistencies in daily practice. The identified inconsistencies primarily arise from efforts to communicate to families that fever itself is not inherently dangerous and that the focus should be on alleviating discomfort. However, a noted contrast exists with a medicalized approach that centers on reducing the child’s temperature.

Participants highlight that these inconsistencies might be apparent to parents when pediatricians lower the temperature as part of routine assessments to determine the child’s baseline status. This action may be driven by established protocols or the necessity to record unaltered vital signs. Additionally, participants note that the history of febrile convulsions can influence the information provided to parents concerning the use of antipyretics. It emphasizes how primary care physicians often initiate discussions about fever during the initial vaccinations of children.

*But for a patient who can wait for you to examine him/her is always better to examine him/her without fever, because what we were talking about, the examination with fever is not going to be real; it is not going to be his/her baseline condition. Of course, and then I always explain to the parents so as not to contradict ourselves: “We have given the child antipyretics so we can examine him/her.”*

H1

*Yes, I believe that the child’s general condition is good even at 39 (°C). It makes no sense to wait for the child’s fever to go down because it will go up again. So, if I tell the child that he/she must lower his/her fever down in the hospital, I think I’m telling him that it’s more serious than it is.*

H6

*Information can be given from the day the child is born, but mainly from the first vaccination because this is when most children face an episode of fever.*

P3

*The most important function would be to provide health education. But we failed radically, radically because instead of getting fewer patients to come, we are getting more. We are making patients dependent.*

P4

*If you administer antipyretic when the temperature starts to rise, then somehow you might be able to stop the seizure.*

P5

*Possibly they would not be discharged with a fever of 39 (°C) because that leads or may lead to a discussion with the parents. What we do is to leave them for an hour to check that this fever has decreased, not because the child requires it, not because we need it, but because discharging a child with a fever often leads to a discussion.*

H2C1

*The child is fine and is doing great. With the antipyretic, we are going to prevent him/her from having a seizure? No, that’s the way it is. We don’t know what’s going to happen to the child, but he/she’s in the hospital, and if anything happens to him/her, we’re here.*

H4C2

*The fever is indeed high! They just gave the child the antipyretic an hour ago. You could let them wait for a while to see if the temperature has come down. Above all, what is important is the general condition of the child.*

P1C1

*Well, tell him that he/she has to act against the fever as if he/she did not have febrile convulsions and that the child will continue to be at risk until he/she is five years old (…) So, no matter how hard you try, you are not going to be able to dismantle this. Because on the first day of diagnosis, you told him/her it was a febrile seizure. And that’s what it’s called.*

P4C2

### 2.11. Relationship with the Evidence

When the participating pediatricians were presented with the recommendations from NICE and AEPED guidelines, they generally agreed with most. However, this category highlights specific points of disagreement with these recommendations. Some participants introduce nuances regarding the use of physical measures. In their professional opinion, they suggest that moderate use of antipyretic therapies might be warranted to alleviate discomfort and can also give parents the perception that they are taking action.

Conversely, the most contentious aspect for some participants revolves around the association between the temperature reached and the severity of infectious diseases. Based on the protocols they follow and their experience, some participants assert that most serious infectious diseases lead to higher temperatures during febrile episodes. However, there are differing opinions, with some participants expressing skepticism about a clear temperature–severity relationship.

*Regarding physical measurements, I can agree, but sometimes, when you must recommend that the family follow certain ways of doing things or instructions, they never like doing nothing. So sometimes they can be recommended, although always with warm water.*

H2

*If the child is more comfortable putting a damp cloth on him/her, why not do it?*

P3

*So it’s one thing if maintaining body temperature is not dangerous or if you don’t have to relate a high body temperature to having a potentially serious bacterial infection. But it is true that most potentially serious infections have a higher fever.*

H1

*It is not the temperature that determines the severity, it is the general condition of the patient when the temperature drops that will tell us if it is a serious, moderate, or severe disease.*

H4

*The greater the likelihood of bacterial infection the higher the fever, especially above 39.5 °C or 40 °C.*

P3

*A high temperature of 40 °C, I believe that there is a consensus that a temperature of 40 °C is a risk factor for serious illness. So here it says that a high temperature is not a risk factor, I’m sorry, but I don’t agree.*

P4

### 2.12. Family Concerns about Fever

This category encompasses family concerns about fever and includes discourses where doctors express observations of behaviors reflecting the family’s fear when seeking health services for their child’s fever episode. Participants empathize with these perceptions, acknowledging that the fear families exhibit is understandable, especially since febrile seizures often serve as catalysts for these fears.

It is noteworthy that certain discourses indicate that the level of empathy a professional feel towards families, whether or not the professional has children themselves, can be influenced and modulated.

Doctors emphasize that families must “do something” when their child has a fever, such as trying to lower the temperature. Doctors acknowledge that it might be appropriate if it helps alleviate parental anxiety. However, they criticize families for frequently resorting to antipyretics as a quick solution and for delegating responsibility to healthcare professionals when seeking assistance with health services.

Primary care pediatricians acknowledge a significant challenge in alleviating family’s fear, referring to it as “fever phobia.” The term suggests a persistent and heightened anxiety or fear related to fever in families.

*And it is logical because when we see a child convulsing, we get upset. And in a hospital, in a hospital environment. So, imagine a parent seeing their child convulsing, it is normal for them to be afraid.*

H1

*I think it is because of fear for the families (we treat the temperature). I think there is a connotation in society that fever is dangerous, and people demand to treat it, that’s why.*

H2

*Some parents come with a temperature record (37.8 °C; 39.1 °C; 39.2 °C) that looks like they have spent the day. That is because they are very worried (…) Many parents say I am not going to let the fever rise because the child is going to have seizures.*

H5

*If you have children, perhaps you empathize differently and you will try to bring the fever down earlier because you are remembering your own experience and you want that parent to feel better.*

H1

*This is due to the fever phobia that exists in society. We pediatricians try to eliminate the phobia that exists. The thing is that it is very difficult. (…) No matter how theoretical you are, you will never be able to remove fear.*

P4

*Well, because they must do something, right? In the same way, sometimes it seems that families come to the emergency room to take responsibility for the pathology. Having a child with a fever and not giving them anything makes them anxious.*

H2

*The thing is, I believe that the treatment that parents do at home is anxiolytic for themselves. They don’t understand that lowering the child’s fever means that the child will go through it a little better. That is, the process that the child is going through will not be modified by the antipyretic. (…) The antipyretic cures everything. (…).*

P1

### 2.13. Impact of the COVID-19 Pandemic

The study interviews took place a year after the COVID-19 pandemic. Physicians noted that children, throughout the pandemic, were well-protected from microorganisms and potential infections. Consequently, they experienced fewer febrile episodes than the usual number of children. This reduction in febrile episodes has changed families’ experiences, making them more concerned when such episodes occur.

Moreover, amid the pandemic, there was a decline in in-person visits to health centers and providing educational workshops for parents by primary care services. This observation was mentioned by emergency pediatricians and supported by primary care pediatricians, possibly contributing to the heightened parental concern regarding febrile episodes.

Apart from these sociocultural factors, the interviewed physicians noted an epidemiological shift since the onset of the pandemic. This shift has resulted in changes in certain properties of other viruses, such as their peak incidence season or the temperature a child reaches when infected.

*And I have the feeling that after the pandemic, Now it has been two years, which is a very long period, without fever, children without masks, children have infected us, so there is a large population that is now very surprised that the child has a fever, and that they are two- or three-year-old children who have not had it and they come.*

H5

*So (during the pandemic) I think that maybe the information is less. Maybe because there hasn’t been as much screening, maybe we’ve skipped primary care centers, maybe phone visits or whatever!*

H5

*Before the pandemic, when everything was a little easier, we did workshops for parents and explained basic childcare things like feeding. We also explained fever and so on. Well, maybe we could go back to this community care that we used to do before and that has now been lost due to the pandemic, and it is going to be difficult to recover.*

P2

*With COVID, we are seeing that it has displaced all the viruses (…) now we are seeing a lot of viruses that have grouped together because they have been displaced by the coronavirus, which causes temperatures, that is, they cause prolonged febrile processes or processes with very high temperatures.*

H1

### 2.14. Inter- and Intra-Professional Relationships

This category encompasses discussions in which pediatricians discuss the practices of other pediatricians, physicians, and nurses regarding fever in children.

A certain tension is evident in the discourse of hospital pediatricians when referring to primary care physicians. Hospital-based pediatricians indicate that the clinical approach may vary depending on the primary care physician attending to the child. They highlight a shortage of pediatricians in health centers and express concerns about family physicians handling pediatric cases, which creates a sense of insecurity for them.

Concerning discourses related to nurses, it is notable how many physicians lack knowledge about the competencies and activities of nurses, expressing their ignorance on the matter. Additionally, pediatricians often characterize nurses as technicians and attribute a maternal role to them. Nevertheless, in the discourses, there is noticeably greater harmony between the doctors and nurses working as a team in primary care.

The interviewees regard professional experience as crucial in managing children with fever, whether medical professionals or nurses. However, it is worth noting that some younger physicians express that more senior physicians may have less current knowledge, highlighting the importance of staying up to date in the field.

*It depends on their primary care pediatrician (…) I sometimes make one or other therapeutic decisions depending on the follow-up of this patient.*

H1

*I mean, I think that there are veteran nurses who tell you that they have had 40 °C. Well, you see, and young nurses who, when they arrive or nurses who are not pediatricians, are very scared.*

H1

*Coordination between the hospital and primary care is essential. Nowadays, it is impossible (…) I think that where you should learn how to take care of a child with a fever is from day 1 in a primary care center.*

H2

*So, if the mother says “oh, the child has a fever”, the nurse goes and takes the temperature. We usually have a prescription for paracetamol or ibuprofen in case the child has a fever and nothing, and it is already prescribed if the child needs it, and if he/she needs it, it is given. If the child has a fever, they give it to him/her.*

H4

*When new pediatricians start out, just like parents and resident family physicians, they tell us that. Well, they come and tell me: “this child has had five hours of fever and has been between 40 °C and 39 °C. They gave him a paracetamol at 14.00 p.m. then they gave him an ibuprofen at 15.00 p.m. Then I tell them: “What use is that to you?”*

H5

*(Do you think that parents are given adequate education from primary care?). I am not sure!*

H6

*I’m sure the nurses don’t know that we ask to lower the temperature so we can assess it more adequately. I think many of them believe that it is necessary to lower the temperature. If there are pediatricians who believe they need to lower the temperature, how can nurses not believe it?*

H6

*The nurse’s role is the same as that of the mother: to assess the general condition of the child. Of course, to explain to the parents the doses of antipyretics and when to give them. In addition, explain to the parents the warning signs and whether it is necessary for the child to be seen by a pediatrician. (…)*

P3

*If you want to analyze it just as a person who does the triage, then it would be a girl who puts the thermometer in and says it’s 37.8 °C. But this is not the function of a nurse. It would have to be triaging from the point of view of observing the person and being able to see the severity.*

P4

*Well, nursing has been gaining more competencies that in principle seem reasonable to me, but I don’t think they should have excessive autonomy either. Medical studies and nursing studies are not the same thing.*

P5

### 2.15. Proposals for Change

This category encapsulates the suggested changes by participants in their daily practice when handling a febrile child. A consensus among most hospital pediatricians is to refrain from administering antipyretics to a sleeping child. However, they still rely on nurses to measure the child’s temperature for documentation. Most hospital pediatricians believe it is essential to enhance the skills of nurses in their units. This improvement should focus on assessing the child’s discomfort rather than solely relying on temperature readings, and they advocate for nurses to have the autonomy to administer antipyretics.

Participants also suggest changes suitable for emergency triage, such as refraining from taking blood glucose readings for feverish children. Primary care pediatricians advocate for the most changes in practice, particularly in enhancing the education provided to families.

*If I have a patient admitted with a serious bacterial illness, I am interested in knowing whether or not he/she has a fever, how much of a fever he/she has, and sometimes even whether or not he/she responds to analgesia. I think that would depend on the pathology. I wouldn’t start by removing temperature measurements. Maybe I would start by removing the analgesia that is given for temperature.*

H2

*(Would you make changes in the guideline that the nurses receive to administer the antipyretic depending on the temperature?) I would leave it that way because it is very difficult to assess. To change it, the nurses would have to see the child with different eyes, let’s say, not only see the guideline and do what the guideline says.*

H3

*(Would you be comfortable with nurses being able to give antipyretic in the hospital autonomously?) Yes. Perfect. As long as we know what was given, and that everything is recorded. Everything that is oral antipyretic, I would agree. But we need to see the fever pattern (…) If the child is sleeping peacefully, you don’t need to wake him/her up to give medication, as that is more uncomfortable.*

H5

*I would make changes in triage. First, not every child with a fever should have all of their vitals taken. Then not every child with a fever should be given something for the fever.*

H6

*Regarding the fact that it is the discomfort and not the fever that is being treated. We should say that a little more so that parents would give antipyretics for this reason, shouldn’t we? And not mistakenly, because of the little number on the thermometer. Of course!*

P2

*Something is wrong, but we don’t know what it is and maybe we should sit down and rethink the health education we are doing. It fails. Let’s go evaluate where it fails because we are unable to transmit it well.*

P3

## 3. Discussion

The current study explores the development of the concept of “childhood fever” as perceived by pediatricians working in both primary care and inpatient pediatric settings.

With this overarching objective, we examined what pediatricians comprehend as fever, the significance of this phenomenon in their day-to-day practice, their therapeutic approaches within their work context, and the connections to existing clinical evidence. Additionally, we investigated how pediatricians perceive the fear of fever among families and how inter-professional and intra-professional relationships among these healthcare professionals are shaped and evolve in response to episodes of fever in children.

Notably, pediatricians respond to the questions and scenarios presented, and they initiate a process of reflection [17] on this routine aspect of their daily practice, particularly episodes of fever. They define fever as a phenomenon that induces frustration due to the high demand it imposes.

Pediatricians draw upon their biochemical knowledge of fever in children and link this phenomenon to the protection and defensive mechanisms of the organism. However, this knowledge is frequently likened to what Freire [18] termed banking education—information acquired but not necessarily applied in the practical management of children with fever in their day-to-day professional routine.

All the physicians in the study have strongly emphasized that treating a febrile child should prioritize addressing discomfort rather than focusing solely on reducing temperature, aligning with established recommendations [8,23]. However, despite their stated intentions, there are observable discrepancies between their ideals and daily practices. Many participants acknowledge these inconsistencies, recognizing a gap between their information and what families may observe.

Doria et al. [14] and similar authors suggest that a potential hindrance to adopting these recommendations is the challenge of evaluating discomfort in children, which may manifest through changes in behavior, mood, and irritability. Additionally, because fever alters baseline constants [23], participants must reduce the temperature to accurately evaluate the child, acknowledging that this can also cause confusion in families.

The recommendations presented to the participants during the interview are not devoid of controversy [8,23]. Among pediatricians, the use of physical measures and the absence of a correlation between the temperature degree and the severity of the infection are particularly contentious.

The participants involved in the study have observed that recommending physical measures is acceptable as long as the intention is to promote the child’s well-being rather than solely lowering the temperature. They also recognize that such measures can give parents a sense of actively caring for their children. This desire of parents to take action is also evident in various qualitative studies involving families [24,25].

In this context, studies evaluating pediatricians’ adherence to recommendations, such as the research conducted by Chiappini et al. [26], reveal that many pediatricians would prescribe physical measures for managing fever in children. It is possible that similar to the findings in our study, pediatricians perceive these measures as capable of alleviating discomfort. Some participants in our study have justified their endorsement by pointing out that the current clinical evidence does not establish contraindications for these measures.

Concerning the association between the temperature degree and the severity of infection, there is disagreement among participants and in the existing literature [27]. A recent systematic review highlights that in children under 3 months old, there is a correlation between temperatures exceeding 40 °C and severe bacterial disease; however, in older children, the temperature degree may not be a significant factor [28].

When questioned about families’ perspectives on fever, pediatricians observed a prevailing sense of apprehension, yet they interpreted these concerns as rational. In this regard, qualitative evidence highlights the need for parents to receive consistent information from professionals that also takes their emotions into account [7].

Notably, febrile seizures emerged as the primary source of worry for families, according to pediatricians. This concern is shared by professionals, including physicians and nurses, as highlighted in a recent systematic review [6]. Despite the awareness that seizures occur in only 2% to 5% of children and are largely benign [9], the fear of seizures remains a commonality between healthcare professionals and families.

It is intriguing that all the pediatricians involved share the perspective that antipyretics do not prevent febrile seizures. Nevertheless, in their discussions or responses to specific cases, they advise parents of children who have experienced these convulsions to monitor temperature more closely and administer antipyretics earlier. These recommendations can be interpreted as defensive actions and anticipatory reactions [19]. Such behaviors aim to mitigate a situation perceived as dangerous or harmful. The interviews revealed a greater prevalence of these actions among primary care pediatricians.

When examining professional relationships in different contexts, we noticed a certain hesitancy among hospital pediatricians regarding primary care. They suggest that families should receive more information about fever from primary care centers. This sentiment aligns with findings from a study conducted in a hospital emergency department waiting room, which concludes that there is no disparity in knowledge among parents coming from primary care versus those coming directly from home. This suggests that they may not have received specific guidance before being referred [29].

In primary care, pediatricians recognize the potential benefits of enhancing the information provided to families regarding fever. However, they acknowledge the existence of a social fever phobia, which impedes the effective transmission of health education. Existing literature identifies parents’ insufficient knowledge in dealing with fever and their perception of a perceived threat as key factors contributing to the prevalence of fever phobia [30].

Primary care pediatricians suggest that one potential area for improvement is the timing of providing initial information about fever to families. The interviewed primary care pediatricians share the viewpoint of physicians in the study conducted by Peetoom et al. [30], indicating that they typically offer this type of education concurrently with the child’s first vaccinations. However, they recognize that this simultaneous approach may lead to confusion among families.

Regarding family education, both inpatient and primary care pediatricians concur that there has been a decline in such education during the COVID-19 pandemic. This decrease may have implications for the knowledge of parents with children born before or during the pandemic, particularly in the care of a child with a fever. This aspect emphasizes the significance of the educational support provided by healthcare centers.

In the inter-professional dynamics involved in managing a child with a fever, it is crucial to consider the various subcultures present within a health service [20]. Of particular significance are the dynamics between physicians and nurses.

The interviewed pediatricians anticipate technical proficiency from nurses [20]. Similar to the perspective of physicians in the studies by Krogstad et al. and Tang et al. [31,32], pediatricians view inter-professional collaboration positively when their indications are executed efficiently and when the patient’s signs and symptoms such as temperature monitoring and administering antipyretics based on specified criteria, are communicated effectively to them.

These attitudes align with the findings of the study by Jeong and Kim [12], where hospital nurses tend to consistently administer antipyretics to febrile children based on pediatricians’ guidelines. However, they may not consider other factors beyond temperature, leading to a perception of a lack of control over the comprehensive management of this symptom.

Nevertheless, the hospital pediatricians involved have reached a consensus on enhancing the capabilities of nurses within their services, enabling them to independently assess whether administering an antipyretic is necessary or not.

Lastly, it is essential to highlight the presence of social learning [19] in the narratives. Pediatricians express how they have acquired many of their behaviors from more experienced professionals within their services, shaping their future approach to fever. Additionally, their role as role models for families is evident [19]. In this context, pediatricians emphasize the necessity for coordination among various services to ensure the provision of consistent information.

On one hand, the study has limitations associated with participant access, constrained by intermediaries or key informants. Additionally, the scarcity of extensive prior literature on this phenomenon constrains the depth of the results and discussion. Furthermore, the absence of qualitative articles with theoretical perspectives hinders the enrichment of the discussion in a similar vein.

The limitations in participant recruitment were successfully addressed, due to the interest shown by certain pediatricians to participate and the implementation of snowball sampling techniques.

The absence of pre-existing theoretical frameworks prompted us to innovate by introducing concepts from authors in the fields of pedagogy and psychology. This approach aimed to enhance the depth of the discussion surrounding the results.

On the other hand, ethnomethodology requires the researcher to understand the lived reality of the participants, and if he or she fails to do so, there is a risk of losing important data. This limitation was solved by including clinical cases in the interviews, through which the participants narrated their daily practice.

The potential limitation of generalizing the results is acknowledged in this study. However, given its qualitative nature, the concept of generalization, understood as the extent to which the findings are applicable to other samples or populations, should be interpreted differently. The results contribute to a deeper understanding of pediatricians’ perspectives on child fever within a specific context. These insights may be transferable to other contexts that share similarities with the settings examined in the study.

## 4. Conclusions

Ethnomethodology has facilitated the exploration of how professionals in primary care and hospital settings manage children with fever as part of their routine.

Pediatricians acknowledge that the approach to fever treatment should prioritize alleviating discomfort rather than solely lowering the temperature. However, they note challenges in implementing these recommendations, including potential conflicts with parents, issues related to service functioning, and complexities in interprofessional relationships.

Pediatricians express willingness to implement specific changes in their daily practice, particularly concerning the administration of antipyretics, with an emphasis on reducing discomfort. They advocate for the increased involvement of nurses and a shift in their role, emphasizing a greater role in patient assessment.

Furthermore, hospital-based pediatricians are calling for a more substantial commitment to parent education regarding fever from primary care, while acknowledging that there is room for improvement in this aspect.

In light of the current situation, it appears fitting to recommend a future research endeavor employing a participatory action research methodology. This study could involve pediatricians and nurses from the studied contexts, aiming to introduce and assess changes in daily practices related to the care of children with fever.

## Figures and Tables

**Figure 1:**
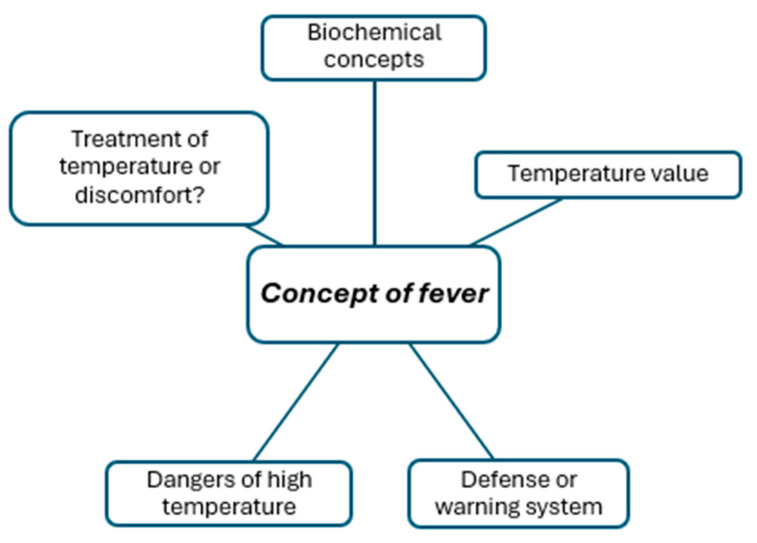
Concept of fever.

**Figure 2:**
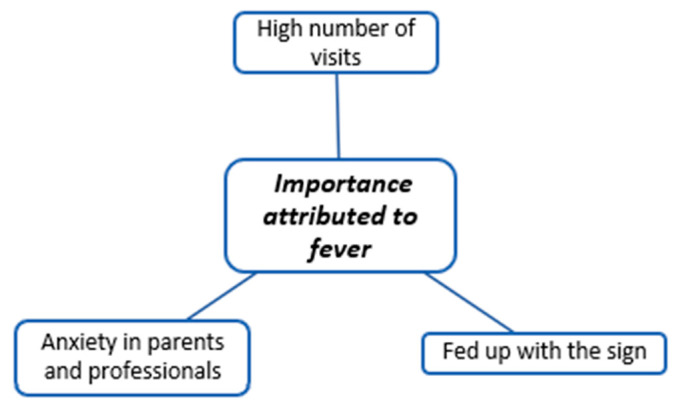
Importance attributed to fever.

**Figure 3:**
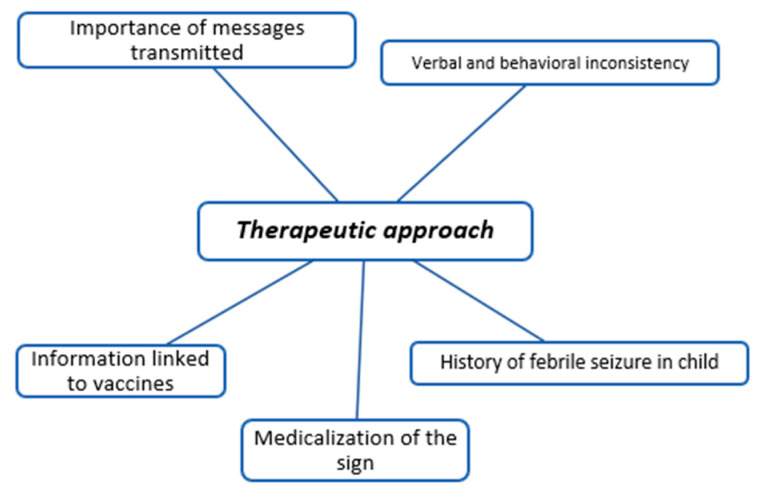
Therapeutic approach.

**Figure 4:**
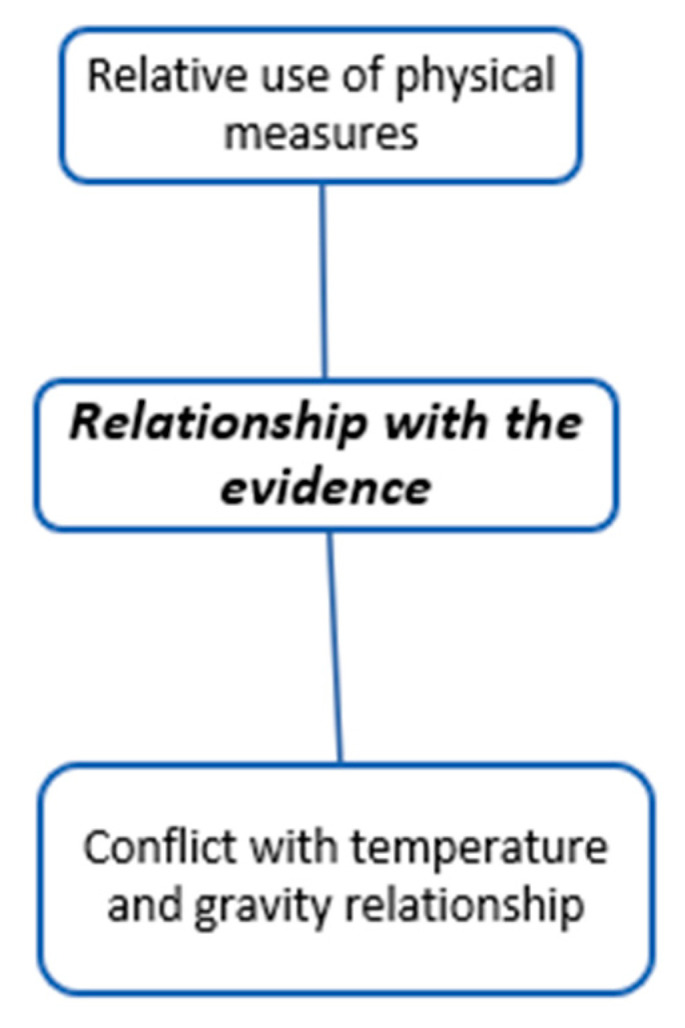
Relationship with the evidence.

**Figure 5:**
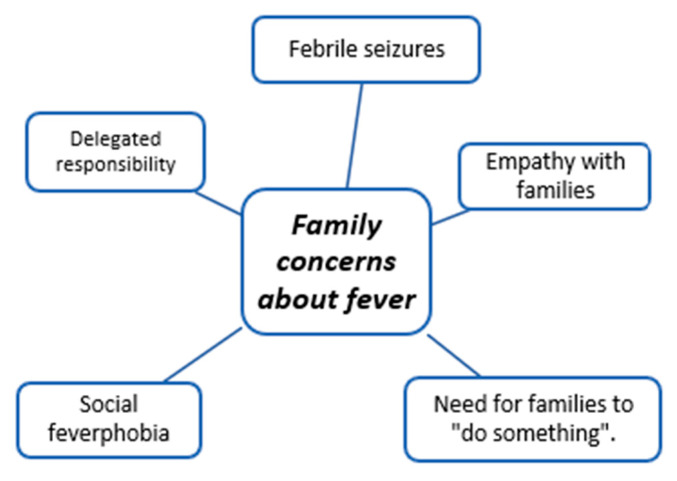
Family concerns about fever.

**Figure 6:**
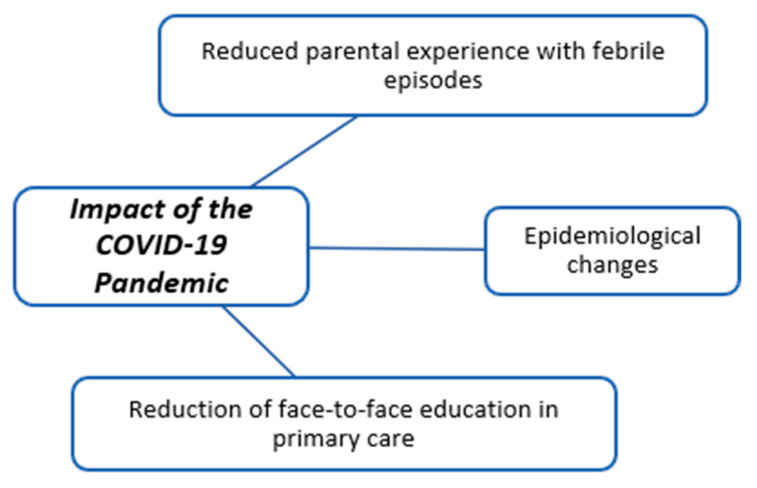
Impact of the COVID-19 Pandemic.

**Figure 7:**
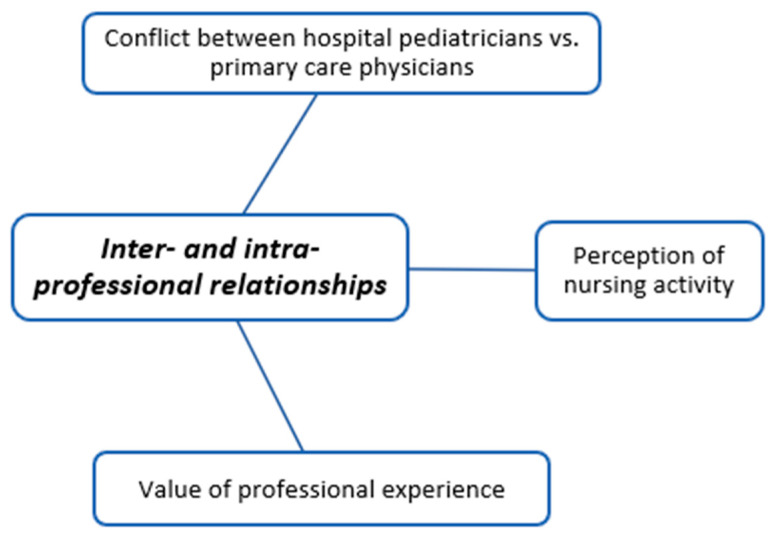
Inter- and intra-professional relationships.

**Figure 8:**
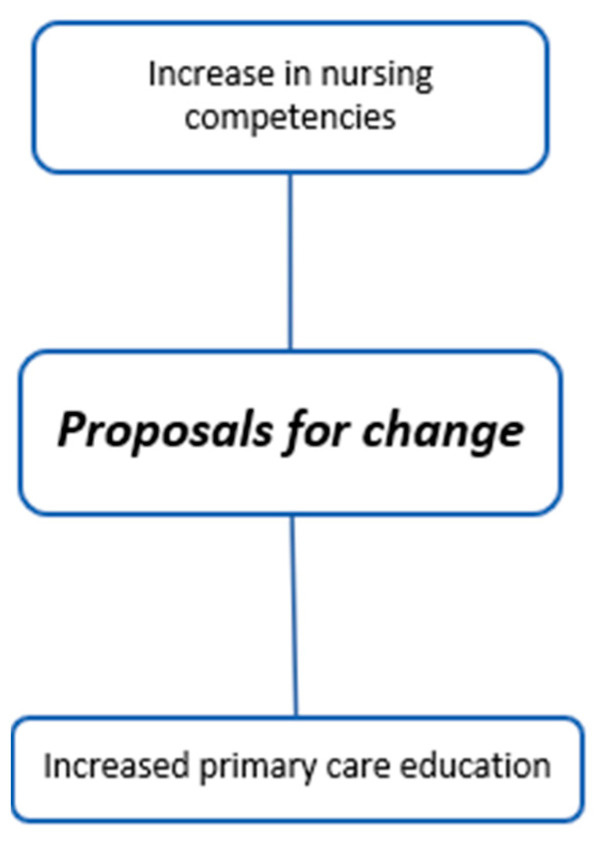
Proposal for change.

**Table 1:** Hospitalization pediatricians’ sociodemographic data.

Primary Care Pediatricians							
Code	Age	Sex	Pediatric Degree	Time Worked in Pediatrics	Sons or Daughters	Member of Scientific Association	Management Position
P1	42	Female	Yes	13 years	No	Yes	No
P2	51	Female	Yes	28 years	Yes	Yes	Yes
P3	59	Female	Yes	34 years	Yes	Yes	No
P4	64	Male	Yes	33 years	Yes	Yes	No
P5	33	Male	Yes	30 years	Yes	Yes	No

**Table 2:** Primary care pediatricians’ sociodemographic data.

Hospitalization Pediatricians (Emergency Room and Inpatient Ward)							
Code	Age	Sex	Pediatric Degree	Time Worked in Pediatrics	Sons or Daughters	Member of Scientific Association	Management Position
H1	32	Female	Yes	8 years	No	Yes	No
H2	36	Male	Yes	11 years	No	Yes	No
H3	56	Female	Yes	20 years	No	Yes	No
H4	53	Female	Yes	30 years	Yes	Yes	No
H5	33	Female	Yes	4 years	No	Yes	No
H6	32	Female	Yes	7 years	No	Yes	No

## Data Availability

The raw data supporting the conclusions of this article will be made available by the authors on request. The data are not publicly available due to privacy or ethical restrictions.

## Supplementary Materials

The following supporting information can be downloaded at: https://www.mdpi.com/article/10.3390/children11030316/s1, Standards for Reporting Qualitative Research (SRQR) [33].
